# Advances in superparamagnetic iron oxide nanoparticles modified with branched polyethyleneimine for multimodal imaging

**DOI:** 10.3389/fbioe.2023.1323316

**Published:** 2024-01-25

**Authors:** Qiaoling Shen, Chunjing Yu

**Affiliations:** ^1^ Department of Nuclear Medicine, Affiliated Hospital of Jiangnan University, Wuxi, China; ^2^ Wuxi School of Medicine, Jiangnan University, Wuxi, China

**Keywords:** BPEI modification, SPION, multimodal imaging, MRI, nanoparticles

## Abstract

Multimodal imaging are approaches which combines multiple imaging techniques to obtain multi-aspect information of a target through different imaging modalities, thereby greatly improve the accuracy and comprehensiveness of imaging. Superparamagnetic iron oxide nanoparticles (SPIONs) modified with branched polyethyleneimine have revealed good biocompatibility and stability, high drug loading capacity and nucleic acid transfection efficiency. SPIONs have been developed as functionalized platforms which can be further modified to enhance their functionalities. Those further modifications facilitate the application of SPIONs in multimodal imaging. In this review, we discuss the methods, advantages, applications, and prospects of BPEI-modified SPIONs in multimodal imaging.

## 1 Introduction

Various molecular imaging techniques such as magnetic resonance imaging (MRI), positron emission tomography (PET), single photon emission computed tomography (SPECT), optical imaging (OI) and ultrasound (US) play a crucial role in the individualized diagnosis and treatment of diseases (Weissleder and Pittet, 2008). These molecular imaging techniques have been applied to evaluate specific molecular targets and visualize the internal structure of the human body. They have also been applied to the non-invasive study of biological processes *in vivo* at the cellular and molecular levels and play a key role in the diagnosis of diseases, patient management, and healthcare. However, among all the current molecular imaging techniques, there is not a single modality that can perfectly provide all the information needed. For example, optical fluorescence imaging is difficult to quantify, and has limited tissue penetration *in vivo*. MRI has high resolution but low sensitivity, while PET offers very high sensitivity but relatively poor resolution (Table 1) (Massoud and Gambhir, 2003; Gleich and Weizenecker, 2005; Alphandéry, 2019; Lu et al., 2021). Therefore, multimodal contrast agents and probes have been developed to solve this problem. Multimodal imaging is the combination of two or more imaging technologies, combining the advantages of different imaging modalities, while minimizing the disadvantages of those technologies. These contrast agents and probes make it possible to visualize, quantify, and trace the molecular processes. They can also detect abnormalities in the human body, obtain new information about some diseases, and achieving the effect of “1 + 1>2” to optimize diagnosis and treatment of diseases. For example, contrast agent and probes can be used to guide the scalpel during surgery (by fluorescence imaging), ensuring that all cancerous materials have been removed (by MRI), and tracking and identifying tumor cells and physiological processes (by PET or SPECT imaging). However, the synthesis of contrast agent and probes poses a huge challenge (Jennings and Long, 2009; Zhou and Brahme, 2010; Misri et al., 2012). Nanoparticles (NPs) have been extensively used as contrast agents for molecular imaging due to their potential in combining multimodal imaging, drug delivery, and targeted therapy into a single entity (Table 2 summarized the current application of advanced nanomaterials for multimodal imaging) (Yuan et al., 2021).

**TABLE 1:** Common imaging techniques and their sensitivity, spatial resolution, temporal resolution, advantages, and disadvantages.

Imaging technique	Used in image generation	Spatial resolution	Temporal resolution	Sensitivity (mole/L)	Depth	Advantages	Disadvantages
PET	High-energy rays	Clinical scanner: 2–3mm; Small animal imaging: sub-millimeter	10 s to minutes	10^–11^–10^−12^	No limit	Provides images of metabolic activity in organisms, valuable for early diagnosis of disease	Use of radiopharmaceuticals with some radiation effects; high costs
SPECT	Low-energy rays	Clinical scanner: ∼3mm; Small animal imaging: sub-millimeter	Minutes	10^–10^–10^−11^	No limit	Provides three-dimensional images of the distribution of radiopharmaceuticals in the body	Lower resolution; long scanning time; use of radiopharmaceuticals
Optical fluorescence imaging	Visible light or near-infrared 2	2–3 mm	Seconds to minutes	Likely 10^−9^–10^−12^	<1 cm	Enables real-time observation inside living organisms and sensitive detection of biomolecules	Limited depth of imaging; high requirements for probe selection
Optical bioluminescence imaging	Visible light	3–5 mm	Seconds to minutes	Not well characterized	1–2 cm	Highest sensitivity, easy, low cost and relative high throughput	Low spatial resolution, current 2D imaging only, relatively surface-weighted, limited translational research
MRI	Radio waves	Clinical scanner: 0.5–1.7mm; Small animal imaging: micron	Minutes to hours	10^−3^–10^−5^	No limit	Provides high-quality soft tissue images; no radiation; multiple viewing angles possible	Long scanning time; high requirements for patient co-operation; sensitivity to metal objects
MPI	Radio frequency	sub-millimeter-millimeter	milliseconds	10^–6^	No limit	No radiation; no penetration depth limit; can combine imaging/therapeutic activity (MPH); large area covered with one scan (can be adjusted by varying coil size)	No widely used; no functional information
PAI	Light	5μm-1 mm	Seconds to minutes	10^−9^–10^–11^	∼5 cm	No radiation; rapid signal acquisition; functional information; good resolution; large covered with one scan	Low penetration depth; not widely used (only prototypes available in clinic)
CT	X-rays	Clinical scanner: sub-millimeter-millimeter; Small animal imaging: micron	Minutes	10^–3^	No limit	Provides detailed images of bone and soft tissue; high resolution; fast operation	The use of X-rays can cause some radiation to the human body
US	High-frequency sound	Clinical scanner: 0.5–1 mm; Small animal imaging: micron	Seconds to minutes	10^−6^–10^–9^	Millimeters to centimeters	No radiation; low cost; real time	Low resolution; high operator skill requirements

MPI: magnetic particle imaging; PAI: photoacoustic imaging; CT: computed tomography.

**TABLE 2:** Current applications of advanced nanomaterials for multimodal imaging.

Multimodal imaging	Imaging technology	Commonly used contrast agent	Advantages	Case	References
Dual-modal Imaging	Fluorescence imaging (FLI) and PAI	Hemoglobin; melanin; indocyanine green; methylene blue; prussian blue; ketonic acid	Photoacoustic imaging integrated with fluorescence improves the targeting and accuracy of *in vivo* imaging	Au-Apt-TPE@Zn HS-CyBz	Chen et al. (2019), Zhang et al. (2019)
	FLI and US	Nanodroplets and gas microvesicles	Improve deep tissue resolution *in vivo*	A fully liquid nanodroplet of hypertonic saline	Chen et al. (2021)
	FLI and MRI	Manganese oxide nanoparticles; gadolinium; magnetic iron oxide nanoparticles	Improve the resolution and sensitivity	P-CyFF-Gd NPs	Yan et al. (2019)
	FLI and PET	Fluorescent molecule;^18^F-FDG;^99m^Tc-HFn	Provide diverse spatial and molecular inferences	PiF	Kang et al. (2020)
	PET/SPECT and CT	^18^F-FDG;^99m^Tc-HFn; gold cluster; iodine gadolinium	High sensitivity for whole-body scanning, a major advantage for tumor therapy assessment	^18^F-RWY; natural H-ferritin nanocages radiolabeled with 99 mTc-HFn	Liang et al. (2018), Xiao et al. (2019)
	PET/SPECT and MRI	^18^F-FDG;^99m^Tc-HFn	Have excellent soft-tissue resolution, high sensitivity and whole-body scanning	99 mTc-DPA-ale-Endorem	Torres Martin de Rosales et al. (2011)
Triple-modal Imaging	FLI, PAI and MRI	Fluorescent molecule; magnetic nanoparticles	Provide more accurate spatial information and display highly efficient	BDPF	Liu et al. (2019)
	FLI, MRI and SPECT	Fluorescent molecule; magnetic nanoparticles; radio-isotope	Fast-screening, quantitative assessment and sufficient sensitivity	m-NCs	Bai et al. (2016)
	FLI, MRI and CT	gold cluster; gadolinium; iodine	Provide more information on the tumor	AuGds	Xu et al. (2017)
	FLI, MRI and US	Magnetic nanoparticles; fluorescent	Importance in the integration of cancer diagnosis and treatment	IR780/Fe_3_O_4_@PLGA/PFP/DOX NPs	Wang et al. (2018)

The application of MRI contrast agents based on magnetic iron oxide nanoparticles began in the 1990s for the clinical diagnosis of liver tumors (Stark et al., 1988). Currently, commercially available products include Feridex (superparamagnetic iron oxide [SPIO]; liver injury imaging), Gastromark (SPIO, gastrointestinal imaging) and Combidex (ultrasmall SPIO [USPIO]; lymphography) (Gao and Yang, 2009). The results of clinical application of these products demonstrate the excellent magnetic imaging performance and *in vivo* safety of magnetic iron oxide nanoparticles in MRI diagnosis. Thus, magnetic iron oxide nanoparticles as MRI contrast agents are a current domestic and international research hotpot. In recent years, a new generation of SPIO nanoparticles (SPIONs) as MRI contrast agents with complex modified structures and functions haven been developed along with the continuous development of nanoparticle preparation technology (Xing et al., 2014; Ajinkya et al., 2020). However, *in vivo* targeted imaging applications place high demands on the physicochemical properties of nanoparticles. SPIONs modified by branched polyethyleneimine (BPEI) exhibit good physicochemical properties and can provide functionalization platforms for further chemical modifications for targeting, drug delivering and other functions (Bao et al., 2014; Molaei et al., 2021).

Here, we briefly summarize the application of SPIONs in multimodal imaging, the synthetic approach to BPEI-modified iron oxide nanoparticles, and the prospects and challenges for their application use in multimodal imaging by searching in the PubMed, Web of Science databases based on the keywords “iron oxide, nanoparticles, polyethyleneimine, multimodal, MRI, *etc.*,”.

## 2 Application of SPIONs in multimodal imaging

MRI is noninvasive, safe, and radiation-free modality and has a high-spatial resolution. Its applications in molecular and cellular imaging are growing rapidly and plays an essential role in diagnosing and staging of tumors. It is not restricted by the penetration depth of the signal, has no ionizing radiation, and exhibits high soft tissue resolution and wide range of clinical applicability. These advantages have made MRI an important imaging technique of clinical tumors diagnosis (Terreno et al., 2010). Since the approval of the first clinical magnetic resonance (MR) contrast agent, namely, Magnevist (Gd-DTPA), by the U.S. Food Surveillance Administration in 1998 up to the present (Boros et al., 2015), the most widely used agent clinically remains to be chelates based on the metal gadolinium (Gd^3+^). Particularly, the demand for gadolinium-based MR contrast agents has been increasing in recent decades, with growing concerns about their safety (Shen et al., 2018). Intravenous administration of gadolinium-based contrast agents (GBCAs) is used due to their ability to reduce T1 and T2 relaxation time. GBCAs are mainly excreted through glomerular filtration, with an excretion half-life of 90 min. Therefore, the potential toxicity of GBCAs is directly related to renal disease. In patients with chronic renal failure, the excretion half-life of GBCAs may be significantly prolonged to 24 h or even longer, which may lead to the retention of GBCAs in the body (Malikova and Holesta, 2017). Studies of the potential clinical implications of gadolinium retention have focused on neurologic and cognitive effects (Habermeyer et al., 2020; Solmaz et al., 2021), as gadolinium can cross the blood-brain barrier and deposit in brain tissue, particularly in the dentate nucleus and basal ganglia (Kanda et al., 2014; McDonald et al., 2015), together with the neurotoxicity of free gadolinium (Rogosnitzky and Branch, 2016). Moreover, intravenous administration of large amounts of GBCAs can result in extensive multiorgan deposition. In a study by Robert J. McDonald et al., healthy rats received 20 intravenous injections of 2.5 mmol gadolinium per kilogram (gadolinium-exposed group) or saline (control group) over a 26-day period. Their results demonstrated that the application of macrocyclic gadolinium chelates instead of linear chelates could reduce the deposition but could not eliminate it (McDonald et al., 2017). Therefore, the development of safe and efficient new contrast agents is of great significance and value. Inorganic nanomaterials, especially magnetic nanoparticles have been extensively studied and applied in the biomedical field (Lutz et al., 2006; Moradi Khaniabadi et al., 2017; Nosrati et al., 2019).

SPIONs are often used as MRI imaging probes in molecular imaging, these particles effectively shorten the T2 of water protons, particularly T2^*^ (Laurent et al., 2008). The mechanism of contrast generation is related to the magnetic properties of nanoparticles, which exert a strong magnetic induction effect on the water protons diffusing around the particles. The relaxation and pharmacological properties of SPIOs are mainly controlled by their size. SPIONs usually consist of a core of magnetite (Fe_3_O_4_) and γ-magnetohematite (Fe_2_O_3_) crystals. A study by Ajay Kumar Gupta et al. concluded that magnetic nanoparticles of 10–100 nm have the best stability and magnetization strength (Gupta and Gupta, 2005). The cores are coated with suitable materials and have total diameters ranging from approximately 60nm–250 nm. Small particles (diameters ranging from 20–50 nm), defined as USPIOs are characterized by a low r2/r1 ratio. Moreover, micrometer-sized particles (micrometer SPIOs) are useful in cell labeling and vascular targeting (Shapiro et al., 2005; Shapiro et al., 2006). Currently, SPIOs are widely used in varieties biomedical applications, such as cell separation (Liu et al., 2008), drug and gene delivery (Pan et al., 2007; Chen et al., 2009; Chen et al., 2010), multimodal imaging photothermal therapy (Cheng et al., 2011), and MRI (Corti et al., 2008; Zhang et al., 2015). For successful biomedical applications, Fe_3_O_4_ NPs are usually required to have good colloidal stability, low nonspecific phagocytosis by the reticuloendothelial system (RES), and active targeting specificity after targeting ligand functionalization. Hence, the surface modifications of Fe_3_O_4_ NPs with hydrophilic and biocompatible polymers are effective and important strategies. Various materials and polymers such as albumin (Berry et al., 2003), dextran (Berry et al., 2004), dendrimers (Shi et al., 2007), polyethylene glycol (PEG) (Kohler et al., 2006), and polyethyleneimine (PEI) (Chertok et al., 2010) are coated on the surface of Fe_3_O_4_ NPs to improve their stability and reduce the clearance by RES.

However, some studies have shown that SPIONs still have many limitations in molecular imaging, such as biocompatibility. Although SPIONs have been widely used in biomedical fields, their biocompatibility is still a problem that needs to be solved. Some studies have also demonstrated that SPIONs may cause toxic reactions in cells, which may impair the normal functions of the cells. Moreover, the stability of SPIONs in organisms is also another issue that requires attention. Under certain conditions, SPIONs may undergo oxidation, aggregation, or decomposition, which may not only hamper their imaging or drug delivery efficacy but may also cause negative implications on organisms. The metabolism of SPIONs in organisms remains another challenge. If SPIONs cannot been efficiently cleared from the organisms, their accumulation in tissues or organs may result in potential health risks. Finally, the preparation of SPIONs is often complicated. It requires precise control of reaction conditions to obtain the desired particle size and shape. Furthermore, surface modifications are also complex and necessary to improve their biocompatibility and functionality (Mahmoudi et al., 2011; Wu et al., 2015; Zhu et al., 2018).

Therefore, researchers have developed a series of surface engineering strategies to modify and functionalize the SPION surface with organic or inorganic materials, such as polymers, biomacromolecules, silica, and metals (Wu et al., 2008; Laurent and Mahmoudi, 2011; Wahajuddin and Arora, 2012). Table 3 summarizes the organic macromolecules which have been used for the iron oxide NPs functionalization and their advantages. Dendrimers are monodispersed polymers characterized by a dendritic structure consisting of oligomers that are repeated and linearly connected through branching units. Dendrimers form macromolecules with dendritic structures through repeated growth and branching, and the degree of branching expands as the number of polymerization generations increases, eventually forming closed three-dimensional spherical structures with embedded cavity structures, surfaces enriched with reactive functional groups, and controllable physicochemical properties. The application of dendrimers for the modification of iron oxide nanoparticles is a novel nanomaterial preparation strategy widely used in the pharmaceutical industry (Sharma et al., 2017). BPEI is a polymer with good water solubility and thermal stability. The branched chains of BPEI are rich in amino groups and have certain internal hydrophobic cavity structures, which provide the potential of multifunctional modification on the surfaces (Li et al., 2016).

**TABLE 3:** Organic macromolecules and their advantages over functionalized iron oxide NPs.

Polymers		Advantages	References
Natural Materials	Dextran	Enables optimum polar interactions with iron oxide surfaces and improves blood circulation time, stability, and biocompatibility	(Berry et al., 2003; Berry et al., 2004; Mikhaylova et al., 2004)
	Starch	Improves biocompatibility and is good for MRI and drug target delivery	(Jie et al., 2019; Krasitskaya et al., 2022)
	Gelatin	Used as a gelling agent and hydrophilic emulsifier; biocompatible	(Olsen et al., 2003; Gaihre et al., 2008)
	Chitosan	Nontoxic, alkaline, and hydrophilic; widely used as nonviral gene delivery system; biocompatible and hydrophilic	(Li et al., 2008a; Li et al., 2008b)
Synthetic Polymers	PEG	Enhances hydrophilicity and water, solubility and improves biocompatibility and blood circulation times	(Paul et al., 2004; Mondini et al., 2008)
	Poly (vinyl alcohol) (PVA)	Prevents agglomeration, giving rise to monodispersibility	(Pardoe et al., 2001; Chastellain et al., 2004; D'Souza et al., 2004)
	Poly (lactide acid) (PLA)	Improves biocompatibility and biodegradability; low toxicity in the human body	(Gomez-Lopera et al., 2006; Chena et al., 2008)
	Alginate	Improves stability and biocompatibility	(Ma et al., 2008; Morales et al., 2008)
	Polymethylmethacrylate (PMMA)	Generally used as thermosensitive drug delivery and cell separation	(Singh et al., 2005; Ling et al., 2017)
	Polyacrylic acid (PAA)	Improves stability and biocompatibility as well as bioconjugation	(Arbab et al., 2003; Shan et al., 2003)
Dendrimers	Polyamidoamine (PAMAM)	Can adjust its chemical properties by changing its surface functional groups and can effectively encapsulate and protect drugs	(Chang et al., 2011; Khodadust et al., 2013; Tajabadi et al., 2013)
	Poly (L-lysine) (PLL)	Strong affinity for cells; can effectively deliver drugs or genes to the inside of cells	(Xiang et al., 2003; Li et al., 2017)
	Poly (propylene) (PPI)	Efficient gene delivery and stability *in vivo*	(Murugan et al., 2018; Far et al., 2020)
	BPEI	Can be chemically modified to improve biocompatibility and reduce toxicity; efficient gene delivery and good *in vivo* stability	(Shi et al., 2007; Chertok et al., 2010; Cai et al., 2013)

## 3 Advantages of BPEI modification of SPIONs

BPEI is a cationic polymer with excellent water solubility and abundant imino and amine groups, and has been used in a wide range of applications (Jager et al., 2012); in particular, BPEI has been used as a modifier for the preparation of composites (Liu et al., 2014) and as delivery vehicles for biomedicine, drug delivery and gene transfection (An and Gao, 2007; Bao et al., 2014). BPEI modification can greatly improve the dispersibility of magnetic nanomaterials Fe_3_O_4_ superparamagnetic nanoparticles, while the surface amine groups enable their conjugations to ligands, antibodies, and drugs. However, the amine groups of BPEI or BPEI-modified nanoparticles may result in severe cytotoxicity and nonspecific cell membrane binding, which may cause undesirable consequence for biological applications. Hence, surface amine group neutralization has been employed to reduce positive charge on surfaces and overcome the above disadvantages. For example, the primary amine groups of BPEI-modified multicarbon nanotubes can be acetylated or carboxylated, which can significantly improve the biocompatibility of those nanotubes (Wen et al., 2013). Table 4 summarizes the advantages of BPEI-modified iron oxide nanomaterials.

**TABLE 4:** Advantages of BPEI-modified iron oxide nanomaterials.

Advantages	
Improved stability	As a cationic polymer, BPEI can stabilize SPIONs by electrostatic adsorption and prevent their aggregation in organisms or during storage
Improved biocompatibility	BPEI modification improves the biocompatibility of SPIONs and reduces their toxicity to cells, making BPEI suitable for use in biomedical applications
Provision of functionalized platforms	BPEI has a large number of amino groups, which can be used as a platform for functionalization; it also provides SPIONs with other functions, such as targeting and drug carrying, through further chemical modification
Improvement of drug loading efficiency	BPEI has a high cation density, which can effectively adsorb and carry negatively charged drugs, thereby improving drug carrying efficiency
Enhanced nucleic acid transfection efficiency	BPEI has a good buffering capacity, which can help SPIONs to pass through the cell membrane and enter the cell interior. Therefore, BPEI-modified SPIONs can be effective transfection vectors for genes or siRNA.

## 4 Methods and applications of BPEI-modified SPIONs

The synthesis methods of BPEI-modified SPIONs include electrostatic adsorption, Covalent binding, Ligand exchange, Hydrothermal method, Photochemistry synthesis and other methods. We summarized the chemical and physical properties as well as applications of different types of BPEI -modified SPIONs synthesis methods (Table 5).

**TABLE 5:** The chemical and physical properties as well as applications of different types of BPEI -modified SPIONs synthesis methods.

Synthesis method	Examples	Molecular weight of BPEI	Application	Hydrodynamic size (nm)	Zeta potential (mV)	SEM/TEM diameters (nm)	Biocompatibility	Stability	References
Electrostatic adsorption	Gal-PEI-SPIOS	---------	Provide target siRNA delivery, achieving oncology treatment	98.2 ± 2.3 nm	+28.51 ± 0.4 mV	∼108 nm	Fe: siRNA>4, cells begin to die	The mixtures of nanoparticles with siRNA protect the siRNA from nuclease degradation beyond 48h	Zhen Yang et al
	Dendrimer modified magnetic iron oxide nanoparticle/DNA/PEI ternary complexes	25 kDa	Increased efficiency of transfection of cos7 cells, a novel strategy for polycation-based *in vitro* gene delivery enhanced by a magnetic field	----------	----------	----------	----------	----------	Wen Ming Liu et al
Covalent binding	BPEI-SPION/pDNA	1800Da	Can rapid and efficient transfection in primary vascular endothelial cells successfully inhibits expression of PAI-1	----------	+2.7 mV	∼50 nm	There was no cytotoxicity observed for any of the BPEI coated SPIONs	BPEI-SPION protect pDNA efficiently against serum enzymes	Ran Namgung et al
	BPEI-TLC-SPION	1800Da	Used as efficient gene delivery carriers that can be tracked by MR.	134.1 nm	8.49 ± 4.82 mV	Discrete particles: 5–10 nm	The lower molecular BPEI 1800Da displayed a reduced cytotoxic effect on the cells compared to BPEI-TLC-SPION	SPIONs have been coated with many polymers for aqueous stability	H. J. Lee et al
						Supra-assembled nanoparticles: 100 nm			
Ligand exchange	scAb_CD3_-PEG-g-PEI-SPION/pDNA	25 kDa	A nonviral vector that effectively transports genes into T cells	106 nm	17 mV	----------	At a higher N/P ratio of 20, the cell viability was significantly lower	----------	Chen Guihua et al
Hydrothermal method	Fe_3_O_4_−PEI	25 kDa	With the proven hemocompatibility and amine conjugation chemistry, maybe applied for various biomedical applications, especially for magnetic resonance imaging and therapy	Can be controlled by varying the mass ratio of Fe (Ⅱ) salt and PEI.			At a relatively low particle concentration, Fe_3_O_4_-PEI with different surface functionalities are pretty healthy	Good colloid stability	Hongdong Cai et al
	Fe_3_O_4_-PEI-FI-HA_6k,_ Fe_3_O_4_-PEI-FI-HA_31k_	25 kDa	a PEI-mediated approach to synthesizing hyaluronic acid (HA)-targeted magnetic iron oxide nanoparticles (Fe_3_O_4_ NPs) for *in vivo* targeted tumor MR imaging applications	190.5nm, 217.1 nm	−16.3 mV	15.6 ± 3.4nm, 16.1 ± 2.9 nm	Both particles are non-cytotoxic at the concentration up to 100mg/mL	Good colloid stability	Jingchao Li et al
					−29.1 mV				
Photochemistry synthesis	MPEG-PEI-SPIONs	Average Mn 400	Have great potential in MRI	34 nm	----------	----------	High biocompatibility	Good stability in water	Yancong Zhang et al
Other methods	Fe_3_O_4_-PEI-RITC	1800Da	Have multimodal MRI-fluorescence imaging and transfection capability	>24.3 ± 5.7 nm	+18.6 mV	24.3 ± 5.7 nm	No adverse effects of the particles on the proliferative capacity of astrocytes	----------	Humphry H. P. Yiu et al
	A-SPIONs	----------	have potential applications in bimodal imaging	13.97 nm	+29.1 mV	9.43 ± 2.93 nm	Have negligible cellular toxicity in SKOV-3, U87-MG, and U251 cell lines	Have high dispersion stability over a broad range of pH, whereas less stability in concentrated NaCl solutions	Donggeon Yoo et al

N/P: the number of nitrogen atoms in delivery agents over that of the phosphate groups in pDNA.

### 4.1 Electrostatic adsorption

**FIGURE 1:**
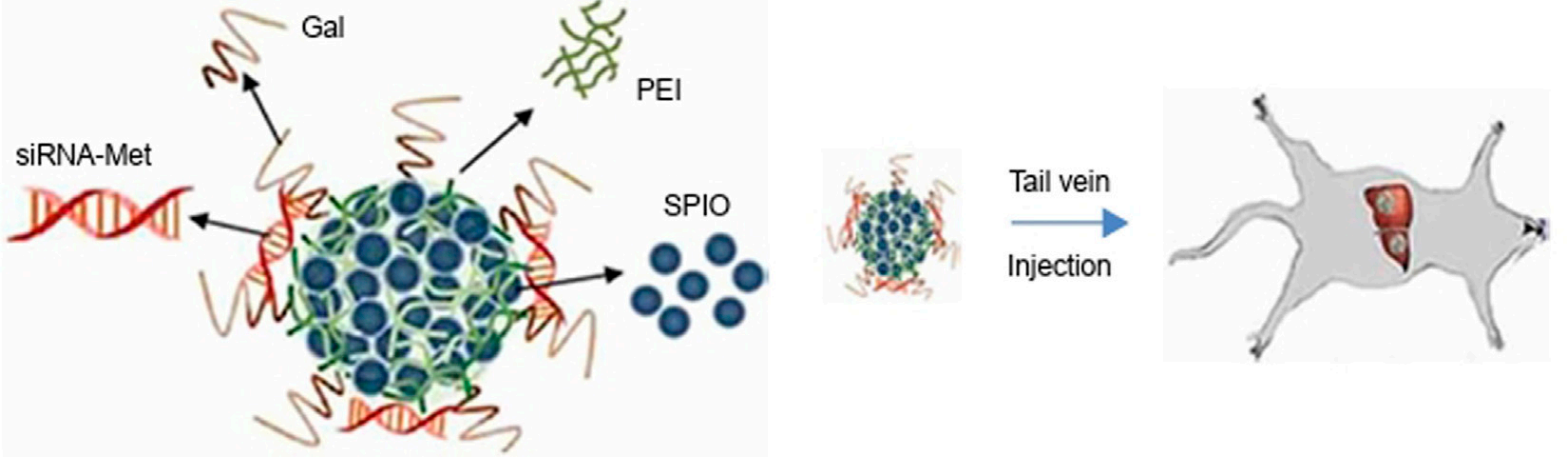
Schematic illustration of Gal-PEI-SPIO nanoencapsulated with siRNA and injected into the mouse (Yang et al., 2018).

SPIO-based nanoparticles are promising platforms for the *in vivo* delivery of siRNA in tumor therapies. The development of novel nanoparticles composed of SPIO provides new options for tumor therapy (Steitz et al., 2007). Through electrostatic interactions, positively charged PEI-coated quantum dots are anchored on the surface of magnetic mandrel, which combine magnetization and efficient fluorescence in tandem for biosensors and clinical diagnostic imaging (Chen et al., 2016). Zhen Yang et al. introduced a novel nanoparticle with a core of iron oxide and modified by galactose (Gal) and PEI, the particle was loaded with siRNA and provided targeted delivery of therapeutic siRNA to liver cancer. The carboxyl-capped Fe_3_O_4_ was initially synthesized using a modified oxidative coprecipitation method, and PEI was further attached to the Fe_3_O_4_-COOH surface by electrostatic adsorption. Finally, Gal-PEG-NH2 was added to the mixed solution to react, and Gal-PEI-SPIOs were purified by removing free PEI and Gal using magnets to precipitate the complex. Gal-PEI-SPIOs, obtained by purification, could tightly bind the siRNA. Gal-PEI-SPIOs could protect siRNA from serum degradation by nuclease in the system, prolong the half-life of siRNA, and deliver the loaded siRNA into tumor cells. Gal-PEI-SPIOs significantly enhance the siRNA accumulation in tumor tissues and inhibited the tumor growth. Gal-PEI-SPIOs provide us with a promising strategy for hepatocellular carcinoma treatment has great prospects in tumor gene therapy (Figure 1) (Yang et al., 2018). Wen Ming Liu et al. reported the use of dendrimer-modified magnetic iron oxide nanoparticle/DNA/PEI ternary complexes for the magnetic infection of mammalian cells. The dendrimer-modified SPION was mixed with plasmid DNA, then cationic polymer PEI was condensed to form ternary complexes with positive surface charges. The results showed that magnetic field significantly increased the transfection efficiency of COS7 cells with the ternary magnetoplexes, particularly in the presence of 10% serum (Liu et al., 2011). Chuanxu Yang et al. also developed a theranostic nanoparticle NP/PEI/siCOX-2 for multimodal imaging and siRNA delivery, which was formed by encapsulation of SPIONs and indocyanine green in a poly (lactic-co-glycolic acid) matrix to serve as a multimodal probe for near-infrared and MRI (Yang et al., 2017).

### 4.2 Covalent binding

**FIGURE 2:**
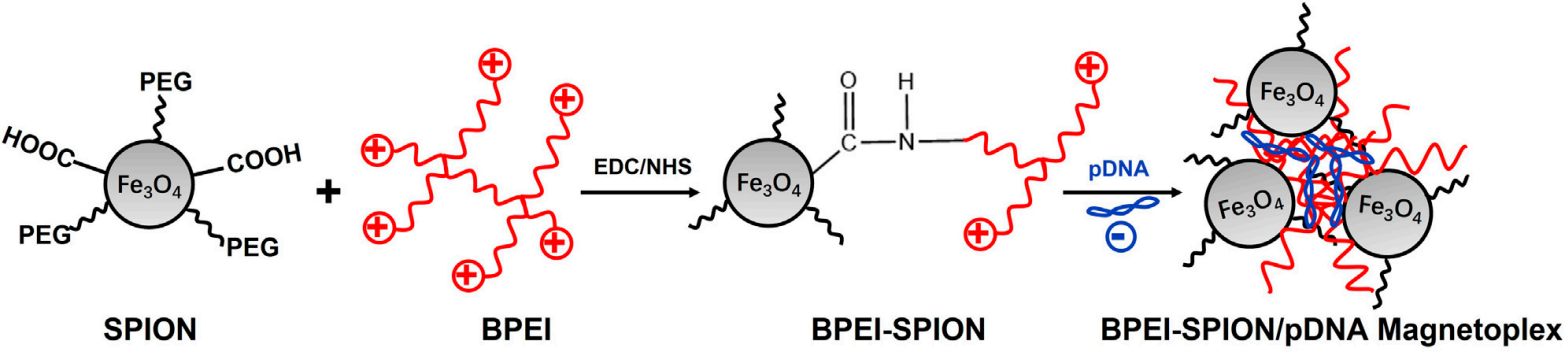
Hybrid BPEI–SPION magnetic nanoparticles.

Most studies on magnetic nanoparticle-mediated transfection have been performed by coating magnetic nanoparticles with cationic polymers, such as BPEI and diethylaminoethyl-dextran (DEAE-dextran). However, the transfection efficiencies of such coated magnetic nanoparticles were not satisfactory. Magnetic nanoparticles attached with PEG molecules and BPEI exhibit excellent magnetic transfection efficiencies even in serum-conditioned media, which enable rapid and efficient transfection of primary vascular cells. PAI-1 plays an important role in various vascular dysfunctions, including vascular inflammation and atherosclerosis. Ran Namgung et al. successfully downregulated PAI-1 expression in primary HUVECs using BPEI-SPION/gWIZ-IL-10, demonstrating the potential of BPEI-SPION as magnetic nanoparticle-mediated targeted gene delivery system (Figure 2) (Namgung et al., 2010). H. J. Lee et al. proposed a strategy of using SPIONs to deliver tumor suppressor genes for tumor therapy, in this study BPEI conjugated thermally cross-linked SPIONs (TCL-SPIONs) were served as a p53 plasmid DNA delivery vehicle. Their results demonstrated that BPEI-TCL-SPIONs successfully delivered p53 plasmid DNA into tumor cells and increased p53 tumor suppressor gene expression. MRI result revealed that the negative contrast enhancement increased in a dose-dependent manner with the increase in the BPEI-TCL-SPIONs concentration in the treated cells. These results indicated that BPEI-TCL-SPIONs could be used as efficient gene delivery carriers and tracked by MRI (Lee et al., 2012). The simple surface functionalization with PEI through glutaraldehyde linker activation gave the complex of PEI-coated Fe_3_O_4_, which loaded isothiocyanate or green fluorescent protein can be visualized and had high transfection efficiency for siRNA and gene delivery (Nguyen et al., 2018).

### 4.3 Ligand exchange

**FIGURE 3:**
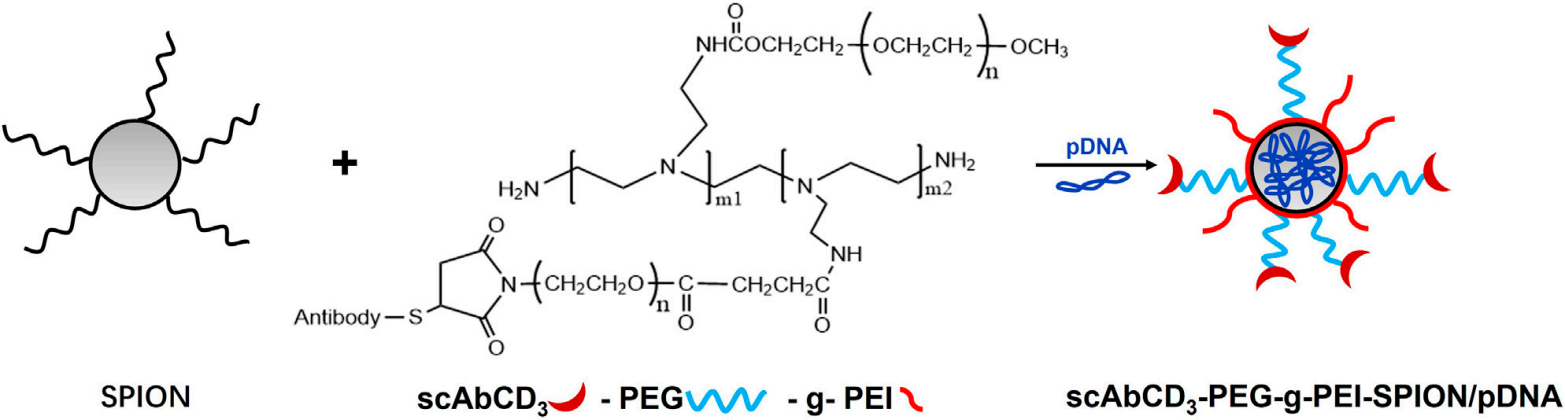
Schematic formation process of magnetic targeting polyplex scAbCD3-PEG-g-PEI-SPION/pDNA.

Nonviral vector-mediated gene therapy has a great advantage over traditional drug therapies in inducing immunosuppression after organ transplantation. Chen Guihua et al. developed a nonviral T cell targeted gene vector by conjugating the T cell specific ligand CD_3_ single-chain antibody (scAbCD_3_) with poly (ethylene glycol)-grafted PEI (scAbCD_3_-PEG-g-PEI). Then scAbCD_3_-PEG-g-PEI polymer was complexed with SPIONs and plasmid DNA was condensed into nanoparticles to form the delivery agent (scAbCD_3_-PEG-g-PEI-SPION/pDNA). Results demonstrated that scAbCD_3_-PEG-g-PEI-SPION/pDNA exhibited not only high gene deliver efficacy but also low cytotoxicity in rat T-lymphocyte line HB8521 cells. Moreover, the targeting effect of scAbCD_3_-PEG-g-PEI-SPION was successfully detected by MRI. This study has proven that scAbCD_3_-PEG-g-PEI-SPION has great potential to be used as a MRI-traceable and T-lymphocyte-targeting gene carrier for immunotherapy (Figure 3) (Chen et al., 2009).

### 4.4 Hydrothermal method

**FIGURE 4:**
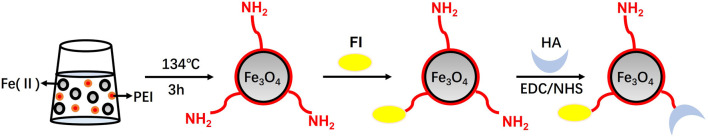
Schematic representation of the synthesis of Fe_3_O_4_-PEI-FI-HA NPs.

Under high-pressure conditions, polymer shrinkage occurs simultaneously with the encapsulation of inorganic nanoparticles in the BPEI branches, and polymer shrinkage increases with pressure. Hongdong Cai et al. reported a simple hydrothermal synthesis and surface functionalization method of BPEI-coated iron oxide nanoparticles (Fe_3_O_4_-PEI NPs). The results demonstrated that the size of Fe_3_O_4_-PEI NPs can be controlled by varying the mass ratio of Fe(II) salts to BPEI. Furthermore, the functionalized Fe_3_O_4_-PEI NPs displayed good aqueous dispersibility, colloidal stability and relatively high R2 relaxivity. The surface PEGylation and acylation endowed the Fe_3_O_4_-PEI NPs with good biocompatibility (Cai et al., 2013). Jingchao Li et al. also reported a BPEI-mediated method of synthesizing hyaluronic acid (HA) targeting magnetic iron oxide nanoparticles for the *in vivo* targeted tumor MRI imaging. HA is an attractive targeting ligand that binds CD44 receptors, which are overexpressed in many kinds of tumor cells. In this work, PEI-Fe_3_O_4_ NPs *via* a one-pot hydrothermal method. The formed PEO-stabilized Fe_3_O_4_ NPs were modified with fluorescein isothiocyanate (FI) and HA with different molecular weight, and finally two kinds of Fe_3_O_4_ NPs were obtained. The researchers demonstrated that HA targeted Fe_3_O_4_ NPs were capable of endocytosis by tumor cells expressing CD44 receptors and serving as targeted MRI probes of cancer cells *in vitro* and xenografts *in vivo* (Figure 4) (Li et al., 2014). More hydrothermal synthesis of nanostructured blends based on SPIONs and branched BPEI polymers have been widely reported (Lv et al., 2014; Popescu et al., 2015).

### 4.5 Photochemistry synthesis

Novel method for synthesis of SPIONs coated with PEI and modified with poly (ethylene glycol) methyl ether (MPEG), MPEG-PEI-SPIONs, was reported by Yancong Zhang et al.. Firstly, Fe3O4 were prepared by co-precipitation method. Then PEI-SPIONs were successfully prepared in aqueous system using photochemical methods and their surfaces were modified with MPEG. T2 relaxation measurements showed that the magnetic resonance signals were significantly enhanced with the increase of the concentration of nanoparticles in water. Therefore, MPEG-PEI-SPIONs have great potential for application in MRI (Zhang et al., 2015).

### 4.6 Other methods

**FIGURE 5:**
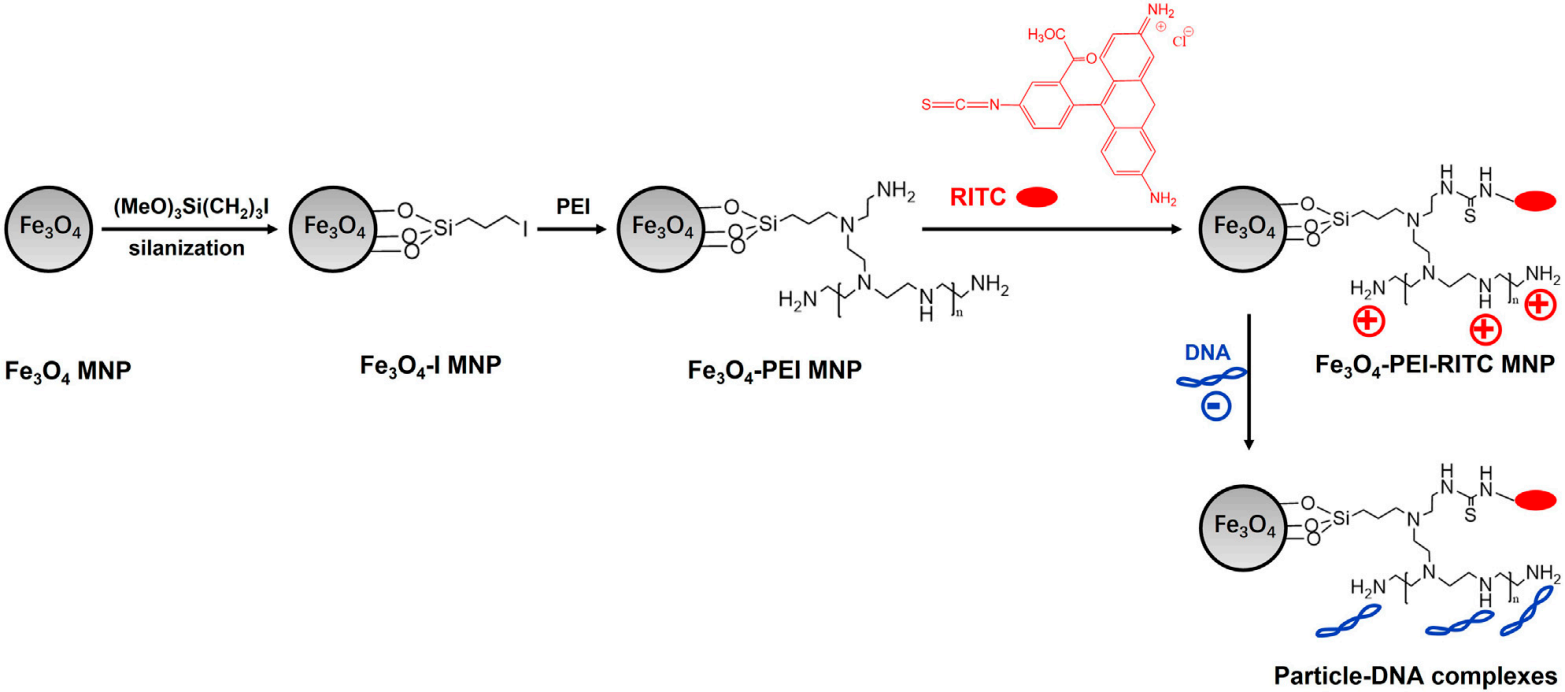
Schematic diagrams of particle synthesis and basic design.

Increasingly methods of BPEI-modified SPIONs are being developed for imaging and therapy (Arsianti et al., 2010; Lentijo Mozo et al., 2017; Mulens-Arias et al., 2019; Zou et al., 2020). Humphrey H. P. Yiu et al. developed Fe_3_O_4_-PEI-RITC magnetic nanoparticles with multimodal MRI- fluorescence imaging and transfection capability, for use in neural cell replacement therapies. The Fe_3_O_4_-PEI-RITC NPs were synthesized by a multi-step chemical grafting procedure: silanisation of NPs with 3-iodopropyltrimethoxysilane; BPEI coupling with iodopropyl groups on the surface and rhodamine isothiocyanate (RITC) binding onto the BPEI coating (Figure 5). The Fe_3_O_4_-PEI-RITC NPs combine MRI and fluorescence imaging capabilities with additional potential for transfection applications, and they can further development for non-invasive cell tracking and gene transfer to neural transplant populations (Yiu et al., 2012). Donggeon Yoo et al. reported the preparation of water dispersible angular-shaped amine-functionalized superparamagnetic iron oxide nanoparticles (A-SPIONs), which synthesized by heating iron (Ⅲ) acetylacetonate in a mixture of solvents containing PEG and BPEI under vigorous stirring. A-SPIONs exhibit high relaxivity for MRI and cyanine 5.5 dye-functionalized A-SPIONs were conducted to investigate their fluorescence imaging applications, which resulted that A-SPIONs have potential applications in multimodal imaging (Yoo et al., 2017).

## 5 Prospects and challenges

Multimodal imaging is a method that combines multiple imaging techniques to provide much more comprehensive and accurate information than single modality. BPEI-modified SPIONs have been used as highly efficient MRI contrast agents, which significantly improve the imaging resolution and contrast. BPEI-modified SPIONs are capable of accommodating different imaging modalities, such as MRI, fluorescence imaging, and photoacoustic imaging, by adjusting their surface properties and structure. While those kinds of modalities have a wide range of prospects for multimodal imaging applications. Apart from the applications in imaging, BPEI-modified SPIONs also demonstrated great potentials of targeted delivery and imaging of specific lesions by conjugating to specific targeting molecules, and these applications greatly facilitate the identification and localization of lesion areas. BPEI modification significantly improves the stability, biocompatibility and biosafety of SPIONs, and reduce the aggregation and clearance *in vivo*. SPIONs exhibit great prospects for applications in medical diagnosis and therapy.

However, challenges and limitations also exist with BPEI-modified SPIONs in multimodal imaging, such as imaging effectiveness, targeting and specificity, stability, and cost-effectiveness. In particular, the design and synthesis of molecular probes with excellent imaging performance are essential challenges. Moreover, the targeting and specificity of nanoparticles in multimodal imaging are vital for precise imaging. Studies have been conducted to achieve localized imaging of specific tissues and cells by introducing specific targeting molecules on the surface of BPEI-modified SPIONs.

Furthermore, the stability of nanomaterials is crucial for long-time imaging and storage. Various strategies have been proposed to improve the stability of BPEI-modified SPIONs, such as the synthesis of stable core-shell structures and the introduction of cross-linking agents. Finally, the cost of nanoparticles’ synthesis and application is also an important concern. Efforts have been conducted to improve the cost-effectiveness of BPEI-modified SPIONs through improving the synthesis methods.

In conclusion, BPEI-modified SPIONs have demonstrated promising applications in multimodal imaging, but further research and improvements are still needed to overcome the existing challenges and limitations, improve the imaging efficacy, targeting, biocompatibility and stability of the nanoparticles through continuously optimization of synthesis methods and surface modification strategies. Through persistent efforts, accurate and reliable multimodal imaging using BPEI-modified SPIONs can be achieved in the future.
